# Hysteroscopic observation of the association between endometrial polyps and endometriosis: a retrospective cohort study

**DOI:** 10.1515/biol-2025-1338

**Published:** 2026-07-07

**Authors:** Hui Liu, Lianhua Tang

**Affiliations:** Obstetrics and Gynecology, Guangzhou Twelfth People’s Hospital, Guangzhou City, Guangdong Province, 510620, China; Gynecology, Guangzhou Eighth People’s Hospital, Guangzhou Medical University, Guangzhou City, Guangdong Province, 510440, China

**Keywords:** endometriosis, hysteroscopy, CA-125 antigen, dysmenorrhoea, infertility

## Abstract

This single-centre retrospective study (100 women with endometrial polyps, mean age 41.8 ± 8.2 years) investigated the association between endometrial polyps and endometriosis. Endometriosis was confirmed in 13 % of patients. Multiple polyps were significantly more common in the endometriosis group (69.2 % vs. 42.5 %, P = 0.046). Women with endometriosis had higher CA125 levels (89.3 ± 45.2 vs. 18.6 ± 12.3 U/mL, P < 0.001) and higher rates of dysmenorrhoea (100 % vs. 35.6 %, P < 0.001). Independent predictors of endometriosis were dysmenorrhoea (aOR[95 % CI] = 8.45 [2.34–30.52], P = 0.001), CA125 > 35 U/mL (aOR[95 % CI] = 12.67 [3.82–42.01], P < 0.001), and multiple polyps (aOR[95 % CI] = 3.12 [1.08–9.01,], P = 0.036). CA125 > 35 U/mL showed an area under the curve (AUC) of 0.852 (95 % CI: 0.778–0.926), and the combined model achieved an AUC of 0.938 (95 % CI: 0.885–0.991) with a bootstrap-corrected AUC of 0.925. During a median follow-up of 17.5 months, polyp recurrence was significantly higher in women with EM (45.5 % vs. 17.6 %, P = 0.047). These findings demonstrate that the combination of multiple polyps, dysmenorrhoea, and elevated CA125 levels should raise suspicion for concurrent EM in women with EPs, suggesting potential shared pathophysiological mechanisms with important implications for clinical management, surgical planning, and fertility counselling.

## 1 Introduction

Endometrial polyps (EPs) and endometriosis (EM) are two of the most common gynaecological conditions in women of reproductive age, with significant implications for fertility and quality of life [1]. A growing body of evidence suggests a significant association between EPs and EM. A recent meta-analysis reported that women with EM have a 2.18-fold higher risk of developing EPs (95 % confidence interval [CI]: 1.58–3.00), particularly in the context of infertility [1]. This finding has been corroborated by multiple independent studies, including an earlier systematic review demonstrating a consistent association across different populations [2], 3]. Furthermore, prospective cohort studies have confirmed that the prevalence of EPs is significantly elevated in women with EM compared to those without, with reported rates ranging from 24 % to 38 % [4], 5]. The consistency of these findings across different study designs and populations strengthens the evidence for a true biological relationship between these two conditions. The pathophysiological mechanisms underlying this association remain incompletely understood, though both conditions share common features including oestrogen dependency, dysregulation of specific inflammatory pathways (e.g. the cyclooxygenase 2/prostaglandin E2 and nuclear factor kappa-light-chain-enhancer of activated B cells signalling pathways), and altered endometrial receptivity [2].

The prevalence of EPs in the general population ranges from 7.8 % to 34.9 %, with higher rates observed in women who are infertile (24 %–32 %) and those with abnormal uterine bleeding [3]. Endometriosis affects approximately 10 %–15 % of women of reproductive age, with peak diagnosis occurring between 30–34 years [1]. The co-occurrence of these conditions presents unique diagnostic and therapeutic challenges, particularly in women seeking fertility treatment. Recent meta-analyses have demonstrated that the association is strongest in women with advanced-stage EM (stages III–IV), with a gradient effect showing higher polyp risk in severe disease (RR 1.19, 95 % CI: 1.08–1.32) [1]. This bidirectional relationship suggests that understanding the factors predicting concurrent disease could improve clinical decision-making and patient outcomes.

Despite growing recognition of the EP–EMs association, several critical knowledge gaps persist. First, the diagnostic accuracy of current imaging modalities and biomarkers for detecting concurrent disease remains suboptimal [4]. By employing the current diagnostic gold standard, that is, combining hysteroscopy, laparoscopy, and histopathology in all cases, the present study was specifically designed to overcome this limitation, providing a reliable basis for evaluating the true association and clinical characteristics of concurrent EP and EMs. While cancer antigen 125 (CA125) has been established as a useful adjunct in diagnosing endometriotic disease, particularly in cystic ovarian EM, as well as deeply infiltrating EM due to its elevation in response to peritoneal inflammation [5], its role in identifying patients with EPs who also have EM has not been systematically evaluated. Second, the impact of this association on fertility outcomes and the optimal management strategy for women with both conditions remains unclear [6]. Third, the potential role of chronic endometritis (CE) as a mediating factor in the EP–EM relationship has emerged as an area of increasing interest, with recent studies suggesting that CE may be present in up to 58.7 % of women with multiple polyps [7].

Furthermore, the temporal dynamics of polyp recurrence in women with EM have not been well characterised. Understanding whether the presence of EM increases the risk of polyp recurrence after hysteroscopic polypectomy could inform surveillance strategies and the need for adjuvant therapies. Additionally, the identification of clinical and biochemical predictors of concurrent disease could help stratify patients who would benefit from comprehensive surgical evaluations, including both hysteroscopy and laparoscopy. Moreover, as the hallmark symptom of EM, dysmenorrhoea may help identify patients with polyps at risk for concurrent disease, though this association remains unexplored.

This study was designed to address these knowledge gaps by systematically evaluating the association between EPs and EM in a well-characterised cohort of women undergoing comprehensive hysteroscopic and laparoscopic evaluation. The primary objective of this retrospective cohort study was to identify clinical and biochemical predictors of EM among women with EPs undergoing hysteroscopic evaluation.

## 2 Methods

### 2.1 Study design and ethical considerations

This single-centre retrospective cohort study was conducted at a tertiary referral centre for reproductive medicine and minimally invasive gynaecologic surgery. The study analysed data from women who underwent surgery between January 2020 and December 2023 (both diagnostic hysteroscopy and laparoscopy). The baseline assessment represented a cross-sectional analysis of factors associated with concurrent EM, while follow-up data enabled cohort analyses of fertility outcomes and polyp recurrence. The study protocol was approved by the institutional review board and conducted in accordance with the Declaration of Helsinki and Good Clinical Practice guidelines. Given the retrospective nature of the study, the requirement for written informed consent was waived by the institutional review board. However, all patients had previously provided general consent for the use of their clinical data for research purposes at the time of hospital admission. As a retrospective cohort study, this design carries inherent risks of selection bias and confounding, which we have addressed through multivariate and sensitivity analyses.

The study followed the Strengthening the Reporting of Observational Studies in Epidemiology guidelines for cohort studies. Data collection was performed by trained research personnel using standardised case report forms, with quality control measures including double data entry and random audits of 10 % of records. Patient confidentiality was maintained through the use of coded identifiers, with the linking key stored separately in a secure, password-protected database accessible only to the principal investigator. All analyses were performed on de-identified data to ensure patient privacy.

**Informed consent:** Given the retrospective nature of the study, the requirement for written informed consent was waived by the institutional review board. All patients had provided general consent for the use of their clinical data for research purposes at the time of hospital admission.

**Ethical approval:** The research related to human use has been complied with all the relevant national regulations, institutional policies and in accordance with the tenets of the Helsinki Declaration, and has been approved by the Research Ethics Committee of Guangzhou Tweifth People’s Hospital (Approval No.: 2025031).

### 2.2 Study population

#### 2.2.1 Inclusion and exclusion criteria

The study population consisted of women of reproductive age (18–50 years) with EPs confirmed by hysteroscopy, who also underwent laparoscopy for comprehensive pelvic evaluation. Inclusion criteria were as follows: (1) aged between 18 and 50 years at the time of surgery; (2) hysteroscopic polypectomy with complete removal of all visible polyps and histopathological confirmation of EP diagnosis; (3) complete hysteroscopic visualization of the uterine cavity with photographic documentation; (4) concurrent laparoscopic evaluation of the pelvis with systematic inspection of the peritoneum, ovaries, and fallopian tubes; (5) histopathological examination of all resected polyp specimens and any endometriotic lesions if present; and (6) availability of pre-surgical CA125 levels drawn within 30 days before the combined hysteroscopy–laparoscopy procedure during the follicular phase (days 3–7) of the menstrual cycle. This comprehensive surgical and histopathological approach represents the reference standard for confirming both EPs and EM, thereby ensuring the accuracy of case classification in our study.

Exclusion criteria included the following: (1) pregnancy or lactation at the time of evaluation; (2) current use of hormonal medications including oral contraceptives, gonadotropin-releasing hormone agonists or antagonists, or aromatase inhibitors within 3 months of surgery; (3) history of pelvic malignancy or radiation therapy; (4) presence of uterine anomalies including unicornuate, bicornuate, or septate uterus; (5) active pelvic inflammatory disease or sexually transmitted infections; (6) previous hysterectomy or bilateral oophorectomy; (7) incomplete surgical or pathological data; (8) submucosal fibroids >2 cm that could confound endometrial assessment; (9) severe intrauterine adhesions (Asherman’s syndrome); and (10) endometrial hyperplasia with atypia or malignancy. These criteria were designed to minimise confounding factors affecting endometrial pathology while ensuring a homogeneous study population.

### 2.3 Data collection

A comprehensive data collection protocol was implemented using standardised case report forms. Baseline demographic data included age, body mass index (BMI), gravidity, parity, and duration of infertility, where applicable. Detailed menstrual history was obtained, including cycle regularity, duration of flow, intermenstrual bleeding, the presence and severity of dysmenorrhoea (assessed using a visual analogue scale from 0–10), and patterns of abnormal uterine bleeding classified according to the PALM (structural: polyp, adenomyosis, leiomyoma, malignancy)-COEIN (non-structural: coagulopathy, ovulatory dysfunction, endometrial, iatrogenic, not classified) system. Previous gynaecologic history included prior surgeries, particularly hysteroscopy or laparoscopy, history of EP removal, previous treatment for EM, and reproductive outcomes of any prior pregnancies.

Clinical symptoms were systematically assessed using validated questionnaires. Dysmenorrhoea severity was categorised as none (Visual Analog Scale [VAS], 0), mild (VAS 1–3), moderate (VAS 4–6), or severe (VAS 7–10). The presence of deep dyspareunia, chronic pelvic pain (defined as non-cyclic pelvic pain lasting >6 months), and bowel or bladder symptoms during menstruation were recorded using standardised symptom scales. Infertility was classified as primary or secondary, with documentation of duration and previous fertility treatments, including ovulation induction, intrauterine insemination, and *in vitro* fertilization attempts. Quality of life assessment was performed using the Endometriosis Health Profile-30 [8] questionnaire when available.

Laboratory investigations included complete blood count with differential, comprehensive metabolic panel, reproductive hormone profile (follicle-stimulating hormone, luteinizing hormone, oestradiol, progesterone, testosterone, anti-Müllerian hormone), thyroid function tests, and tumour markers. Cancer antigen 125 levels were measured using a standardised chemiluminescent immunoassay (Roche Diagnostics, Basel, Switzerland) with results expressed in U/mL. Carbohydrate antigen 19–9, when available, was also recorded. All laboratory tests were performed in the follicular phase (days 3–7) of the menstrual cycle to minimise cyclic variations. Additional inflammatory markers, including C-reactive protein and erythrocyte sedimentation rate, were recorded when available.

### 2.4 Main outcome measures

#### 2.4.1 Primary outcome

The primary outcome was the presence of EM among women with confirmed EPs. Endometriosis was defined as visual identification of endometriotic lesions during laparoscopy with histopathological confirmation showing endometrial glands and stroma. This binary outcome served as the dependent variable in our predictive models, with the goal of identifying which clinical and biochemical factors could predict concurrent EM in women presenting with EPs. Multiple polyps were defined as the presence of two or more discrete polyps within the uterine cavity.

#### 2.4.2 Secondary outcomes

Secondary outcomes included the following: (1) polyp characteristics including number (single vs. multiple, with multiple defined as ≥2 polyps), maximum diameter measured in millimetres, location within the uterine cavity (fundus, anterior wall, posterior wall, lateral walls, cornual regions), and morphology (pedunculated vs. sessile, based on the angle of attachment <90° vs. ≥90°); (2) EM staging according to the revised American Society for Reproductive Medicine classification system: Stage I (minimal, 1–5 points), Stage II (mild, 6–15 points), Stage III (moderate, 16–40 points), and Stage IV (severe, >40 points); (3) the presence of CE, defined as ≥5 Syndecan-1 (CD138)-positive plasma cells per high-power field (HPF) in endometrial biopsy specimens; (4) pregnancy outcomes in women attempting conception during the follow-up period, including clinical pregnancy rate, miscarriage rate, and live birth rate; (5) polyp recurrence was defined as the detection of new EPs by hysteroscopy or transvaginal ultrasound during follow-up after complete polypectomy; polyp recurrence rate defined as hysteroscopic or ultrasound detection of new polyps during follow-up; and (6) time to polyp recurrence analysed using survival analysis methods.

### 2.5 Sample size calculation

Sample size calculation was performed using G*Power 3.1 software (Heinrich Heine University Düsseldorf, Düsseldorf, Germany). Given that our primary analysis involved predicting EM (binary outcome) among women with EPs using logistic regression, we based our calculation on the events per variable (EPV) rule and the expected prevalence of EM. Previous studies suggested that approximately 13 %–20 % of women with EPs have concurrent EM [9]. With an expected EM prevalence of 15 % and planning to evaluate up to 3 predictor variables in the final multivariate model to maintain an EPV ≥5, we required a minimum of 100 patients. This sample size would provide 80 % power to detect an odds ratio of 3.0 or greater for key predictors at a two-sided significance level of 0.05. Additionally, this sample size would allow for adequate precision in estimating the area under the curve (AUC) for diagnostic accuracy assessment, with an expected standard error of approximately 0.05 for an AUC of 0.85.

We enrolled 100 consecutive eligible patients meeting all of the inclusion criteria. Although 100 was the calculated minimum, our strict inclusion criteria (requiring both hysteroscopy and laparoscopy with complete data) limited the number of eligible patients during the study period, making 100 consecutive patients the full available cohort.

### 2.6 Follow-up protocol

Follow-up data were collected through the review of medical records and telephone interviews when necessary. The follow-up protocol included the following: (1) routine postoperative visit at 4–6 weeks; (2) ultrasound evaluation at 6 months and annually thereafter for polyp recurrence surveillance; (3) documentation of pregnancy attempts and outcomes; (4) recording of any additional gynaecologic procedures; and (5) assessment of symptom resolution or recurrence. For fertility outcomes, we recorded time to pregnancy from surgery, method of conception (spontaneous vs. assisted), and pregnancy outcomes (clinical pregnancy, miscarriage, live birth). For polyp recurrence, we documented the method of detection (ultrasound vs. hysteroscopy), time to recurrence, and management approach. Patients were censored at the date of their last follow-up, pregnancy (for recurrence analysis), or December 31, 2023, whichever came first. The median follow-up duration and loss to follow-up rates are reported in the results section.

### 2.7 Statistical analysis

Statistical analysis was performed using SPSS version 26.0 (IBM Corp., Armonk, NY, USA) and R version 4.1.0 (R Foundation for Statistical Computing, Vienna, Austria). The R software was specifically used for penalised regression analyses and bootstrap validation. Bootstrap resampling with 1,000 iterations was performed for internal validation of the predictive model, providing optimism-corrected estimates of the AUC and calibration slopes to assess overfitting. Continuous variables were assessed for normality using the Shapiro–Wilk test and presented as mean ± standard deviation for normally distributed data or median (interquartile range [IQR]) for non-normally distributed data. Categorical variables were expressed as frequencies and percentages. Group comparisons were performed using independent *t*-tests or a Mann–Whitney *U* test for continuous variables, and a chi-square or Fisher’s exact test for categorical variables, as appropriate. Given the relatively small number of EM events (*n* = 13), we employed Firth’s penalised logistic regression to address potential small-sample bias and separation issues. Firth’s penalised logistic regression was employed to reduce small-sample bias by maximizing a penalised likelihood function, which is particularly suitable for datasets with rare events (*n* = 13) to avoid separation issues and provide more stable estimates. Univariate analyses were first conducted to identify factors associated with EM. Variables with P < 0.10 in univariate analysis were considered for inclusion in the multivariate model. To avoid overfitting, we limited the final multivariate model to three predictor variables, maintaining an EPV >4. Model selection was performed using backward stepwise selection with a removal criterion of P > 0.05. Odds ratios (ORs) with 95 % confidence intervals (CIs) were calculated using both standard and penalised methods, with the penalised estimates reported in the final results. The discriminatory ability of CA125 and other biomarkers was assessed using receiver ROC curve analysis, with calculation of the AUC, sensitivity, specificity, positive predictive value (PPV), and negative predictive value (NPV). The Youden index was used to determine optimal cutoff values. Bootstrap resampling (1,000 iterations) was performed for internal validation of the predictive model, with calculation of the optimism-corrected AUC and calibration slopes. Calibration was assessed using calibration plots comparing predicted versus observed probabilities. For time-to-event outcomes (pregnancy and polyp recurrence), Kaplan–Meier survival analysis was performed with log-rank tests for group comparisons. Cox proportional hazards regression was used to calculate hazard ratios. Patients were censored at the date of last follow-up if the event had not occurred. The proportional hazards assumption was tested using Schoenfeld residuals. Sensitivity analyses were conducted to assess the robustness of the findings, including: (1) analyses restricted to women with histologically confirmed EM, excluding those with only adenomyosis; (2) analyses using different CD138 cutoff values (≥1, ≥5, ≥10 cells/HPF) for CE diagnosis; (3) analyses excluding postmenopausal women; and (4) analyses using multiple imputation for missing laboratory values. Statistical significance was set at P < 0.05 for all primary analyses. For secondary and exploratory analyses, we report nominal P-values without adjustment for multiple comparisons but acknowledge the increased risk of Type I error. All P-values are two-tailed. Given the exploratory nature of secondary analyses, nominal p-values are reported without adjustment for multiple comparisons; however, the risk of Type I error should be considered when interpreting these findings.

## 3 Results

### 3.1 Baseline characteristics

The study cohort comprised 100 women with histologically confirmed EPs who underwent both a hysteroscopy and a laparoscopy. The mean age was 41.8 ± 8.2 years (range 26–50 years). Among them, 13 women (13.0 %) had confirmed EM, including 6 with ovarian endometriomas, 4 with adenomyosis confirmed by imaging and histopathology, and 3 with peritoneal EM. Among those with peritoneal disease, lesions were predominantly located in the pouch of Douglas and uterosacral ligaments. The demographic and clinical characteristics stratified by EM status are presented in Table 1.

**Table 1:** Baseline demographic and clinical characteristics.

Characteristic	Total (*n* = 100)	Endometriosis (*n* = 13)	No endometriosis (*n* = 87)	*p*-Value
**Demographics**				
Age (years), mean ± SD	41.8 ± 8.2	32.5 ± 5.4	43.2 ± 7.8	<0.001
BMI (kg/m^2^), mean ± SD	24.6 ± 3.8	22.1 ± 2.9	25.0 ± 3.9	0.012
**Reproductive history**				
Gravidity, median (IQR)	1 (0–2)	0 (0–1)	1 (0–2)	0.023
Parity, median (IQR)	1 (0–1)	0 (0–0)	1 (0–2)	0.008
Nulliparity, n (%)	42 (42.0)	10 (76.9)	32 (36.8)	0.006
Primary infertility, n (%)	32 (32.0)	8 (61.5)	24 (27.6)	0.028
Duration of infertility (years), mean ± SD	3.2 ± 2.1	4.8 ± 2.3	2.9 ± 1.9	0.003
**Menstrual characteristics**				
Regular cycles, n (%)	77 (77.0)	9 (69.2)	68 (78.2)	0.485
Cycle length (days), mean ± SD	28.5 ± 3.2	27.8 ± 2.9	28.6 ± 3.3	0.412
Flow duration (days), mean ± SD	5.2 ± 1.8	6.1 ± 2.0	5.0 ± 1.7	0.041
Heavy menstrual bleeding, n (%)	45 (45.0)	9 (69.2)	36 (41.4)	0.058
**Clinical symptoms**				
Dysmenorrhea, n (%)	44 (44.0)	13 (100.0)	31 (35.6)	<0.001
– Mild	16 (16.0)	2 (15.4)	14 (16.1)	
– Moderate	18 (18.0)	4 (30.8)	14 (16.1)	
– Severe	10 (10.0)	7 (53.8)	3 (3.4)	
Abnormal uterine bleeding, n (%)	58 (58.0)	8 (61.5)	50 (57.5)	0.780
Chronic pelvic pain, n (%)	21 (21.0)	9 (69.2)	12 (13.8)	<0.001
Deep dyspareunia, n (%)	15 (15.0)	6 (46.2)	9 (10.3)	0.003
Bowel symptoms during menses, n (%)	12 (12.0)	5 (38.5)	7 (8.0)	0.007
**Previous surgery**				
Prior hysteroscopy, n (%)	5 (5.0)	1 (7.7)	4 (4.6)	0.508
Prior laparoscopy, n (%)	8 (8.0)	3 (23.1)	5 (5.7)	0.065
Prior polyp removal, n (%)	3 (3.0)	1 (7.7)	2 (2.3)	0.336

Women with EM were significantly younger (32.5 vs. 43.2 years, *P* < 0.001), had a lower BMI (22.1 vs. 25.0 kg/m^2^, P = 0.012), and were more likely to be nulliparous (76.9 % vs. 36.8 %, P = 0.006). Primary infertility was more prevalent in the EM group (61.5 % vs. 27.6 %, P = 0.028), with a longer mean duration of infertility (4.8 vs. 2.9 years, P = 0.003). All women with EM experienced dysmenorrhoea compared to only 35.6 % of those without EM (P < 0.001), with 53.8 % reporting severe symptoms.

### 3.2 Polyp characteristics and distribution

The morphological characteristics of EPs differed significantly between women with and without EM (Table 2). Multiple polyps were significantly more common in women with EM (69.2 % vs. 42.5 %, P = 0.046), with an OR of 3.05 (95 % CI: 1.02–9.12). The distribution of polyp number showed that 30.8 % of women with EM had 4 or more polyps compared to only 14.9 % of those without EM.

**Table 2:** Endometrial polyp characteristics by endometriosis status.

Polyp characteristics	Endometriosis (*n* = 13)	No endometriosis (*n* = 87)	*p*-Value	Or (95 % CI)
**Polyp number**				
Single	4 (30.8)	50 (57.5)		Reference
2–3 polyps	5 (38.5)	24 (27.6)	0.038	2.60 (0.63–10.78)
≥4 polyps	4 (30.8)	13 (14.9)	0.085	3.85 (0.83–17.81)
Multiple polyps (≥2)	9 (69.2)	37 (42.5)	0.046	3.05 (1.02–9.12)
**Maximum diameter**				
<10 mm, n (%)	3 (23.1)	35 (40.2)		
10–20 mm, n (%)	7 (53.8)	38 (43.7)	0.412	0.61 (0.52–4.99)
>20 mm, n (%)	3 (23.1)	14 (16.1)	0.678	1.79 (0.45–7.11)
Mean diameter (mm)	14.2 ± 6.8	12.8 ± 7.2	0.516	
**Location**				
Fundus, n (%)	2 (15.4)	18 (20.7)	0.736	0.70 (0.14–3.42)
Anterior wall, n (%)	4 (30.8)	23 (26.4)	0.745	1.24 (0.35–4.36)
Posterior wall, n (%)	6 (46.2)	35 (40.2)	0.683	1.28 (0.40–4.08)
Lateral walls, n (%)	3 (23.1)	15 (17.2)	0.696	1.44 (0.35–5.89)
Cornual region, n (%)	5 (38.5)	12 (13.8)	0.041	3.89 (1.14–13.28)
Multiple locations, n (%)	8 (61.5)	28 (32.2)	0.036	3.38 (1.08–10.56)
**Morphology**				
Pedunculated, n (%)	5 (38.5)	42 (48.3)		Reference
Sessile, n (%)	4 (30.8)	28 (32.2)	0.564	1.20 (0.29–4.94)
Mixed types, n (%)	4 (30.8)	17 (19.5)	0.328	1.98 (0.46–8.49)
**Vascularity pattern**				
No visible vessels	2 (15.4)	25 (28.7)		Reference
Single feeding vessel	6 (46.2)	38 (43.7)	0.472	1.97 (0.37–10.58)
Multiple vessels	5 (38.5)	24 (27.6)	0.232	2.60 (0.46–14.73)
**Hysteroscopic features**				
Surface irregularity	7 (53.8)	28 (32.2)	0.121	2.46 (0.78–7.74)
Cystic appearance	3 (23.1)	11 (12.6)	0.382	2.07 (0.50–8.60)
Hemorrhagic areas	4 (30.8)	14 (16.1)	0.244	2.31 (0.62–8.61)

**Figure 1:**
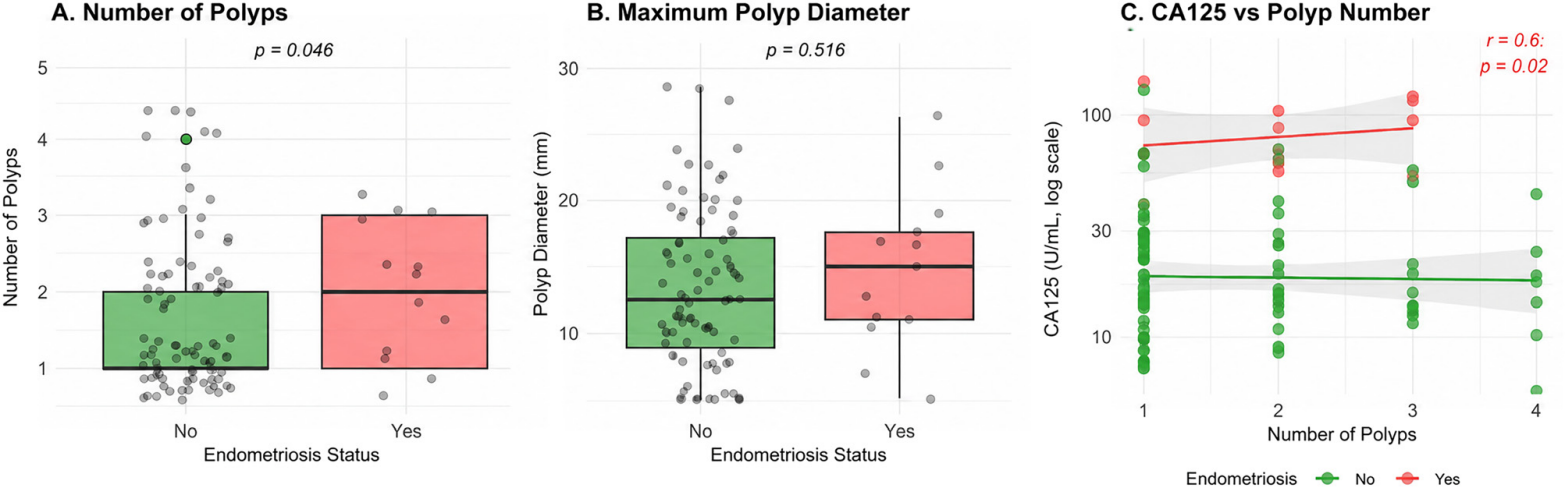
Distribution of polyp characteristics by endometriosis status. (A) Number of polyps in patients with (*n* = 13) and without (*n* = 87) endometriosis. (B) Maximum polyp diameter by endometriosis status. (C) Correlation between CA125 levels and polyp number stratified by endometriosis. Boxes show median and IQR; p-values from Mann-Whitney *U* test or spearman correlation.

Polyps located in the cornual regions were more frequent in the EM group (38.5 % vs. 13.8 %, P = 0.041), as were polyps involving multiple locations within the uterine cavity (61.5 % vs. 32.2 %, P = 0.036). The mean maximum polyp diameter did not differ significantly between groups (14.2 vs. 12.8 mm, P = 0.516), suggesting that polyp multiplicity rather than size was the distinguishing feature (Figure 1).

### 3.3 Laboratory findings and biomarkers

Laboratory parameters showed marked differences between women with and without EM (Table 3). The CA125 levels were significantly elevated in women with EM (89.3 ± 45.2 vs. 18.6 ± 12.3 U/mL, P < 0.001). The proportion of patients with CA125 > 35 U/mL was 76.9 % in the EM group compared to 10.3 % in the non-EM group (P < 0.001).

**Table 3:** Laboratory parameters and biomarker levels.

Laboratory parameter	Endometriosis (*n* = 13)	No endometriosis (*n* = 87)	*p*-Value	AUC (95 % CI)
**Hematological**				
Hemoglobin (g/L), mean ± SD	118.5 ± 15.2	122.3 ± 18.6	0.491	–
Anemia (<110 g/L), n (%)	3 (23.1)	18 (20.7)	0.728	–
WBC (×10^9^/L), mean ± SD	6.8 ± 2.1	6.4 ± 1.9	0.492	–
Platelets (×10^9^/L), mean ± SD	245 ± 62	238 ± 58	0.689	–
**Tumor markers**				
CA125 (U/mL), mean ± SD	89.3 ± 45.2	18.6 ± 12.3	<0.001	0.852 (0.778–0.926)
CA125 > 35 U/mL, n (%)	10 (76.9)	9 (10.3)	<0.001	–
CA125 > 65 U/mL, n (%)	7 (53.8)	2 (2.3)	<0.001	–
CA19-9 (U/mL), mean ± SD^a^	28.4 ± 15.3	22.1 ± 11.2	0.156	0.612 (0.482–0.742)
**Hormonal profile**				
FSH (IU/L), mean ± SD	7.2 ± 2.8	8.1 ± 3.4	0.374	–
LH (IU/L), mean ± SD	5.8 ± 2.2	6.3 ± 2.9	0.559	–
E2 (pg/mL), mean ± SD	48.3 ± 18.6	45.2 ± 20.1	0.604	–
Progesterone (ng/mL), mean ± SD	0.8 ± 0.4	0.9 ± 0.5	0.512	–
AMH (ng/mL), mean ± SD	3.2 ± 1.8	2.8 ± 2.1	0.521	–
**Inflammatory markers**				
CRP (mg/L), median (IQR)^b^	3.8 (2.1–6.2)	2.9 (1.8–4.5)	0.234	0.589 (0.413–0.765)
ESR (mm/hr), mean ± SD^b^	18.5 ± 8.2	15.2 ± 7.6	0.189	0.601 (0.428–0.774)
Neutrophil/lymphocyte ratio	2.8 ± 1.2	2.4 ± 1.0	0.198	–

^a^Data available for 68 patients; ^b^Data available for 52 patients.

The ROC analysis revealed that CA125 had good diagnostic accuracy for predicting EM with an AUC of 0.852 (95 % CI: 0.778–0.926). Using a cutoff of 35 U/mL, CA125 had a sensitivity of 76.9 %, specificity of 89.7 %, PPV of 52.6 %, and NPV of 96.3 %. The optimal cutoff determined by the Youden index was 42 U/mL, yielding a sensitivity of 69.2 % and specificity of 91.9 %.

### 3.4 Association with chronic endometritis

The prevalence and characteristics of CE differed significantly between women with and without EM (Table 4). Syndecan-1 immunohistochemistry was performed in 83 % of patients as part of routine endometrial evaluation.

**Table 4:** Chronic endometritis and associated findings.

Parameter	Endometriosis (*n* = 13)	No endometriosis (*n* = 87)	*p*-Value
**CD138 immunohistochemistry**			
CD138 performed, n (%)	11 (84.6)	72 (82.8)	1.000
CD138 positive (≥5 cells/HPF), n (%)	7/11 (63.6)	18/72 (25.0)	0.015
CD138 positive (≥1 cell/HPF), n (%)^a^	9/11 (81.8)	32/72 (44.4)	0.027
CD138 positive (≥10 cells/HPF), n (%)^a^	4/11 (36.4)	8/72 (11.1)	0.048
Mean CD138 count/HPF	8.2 ± 4.3	3.6 ± 2.8	<0.001
**Hysteroscopic features of CE**			
Micropolyps, n (%)	5 (38.5)	12 (13.8)	0.041
Stromal edema, n (%)	6 (46.2)	15 (17.2)	0.024
Focal hyperemia, n (%)	8 (61.5)	21 (24.1)	0.009
Strawberry appearance	3 (23.1)	7 (8.0)	0.117
**Combined CE findings**			
CE by any criteria, n (%)	9 (69.2)	25 (28.7)	0.005
CE + multiple polyps, n (%)	6 (46.2)	8 (9.2)	0.002
CE + dysmenorrhea, n (%)	9 (69.2)	11 (12.6)	<0.001
**Microbiological assessment^b^**			
Bacterial culture performed	8 (61.5)	45 (51.7)	0.506
Positive culture	3/8 (37.5)	8/45 (17.8)	0.336

^a^Sensitivity analysis with different thresholds; ^b^When clinically indicated.

**Figure 2:**
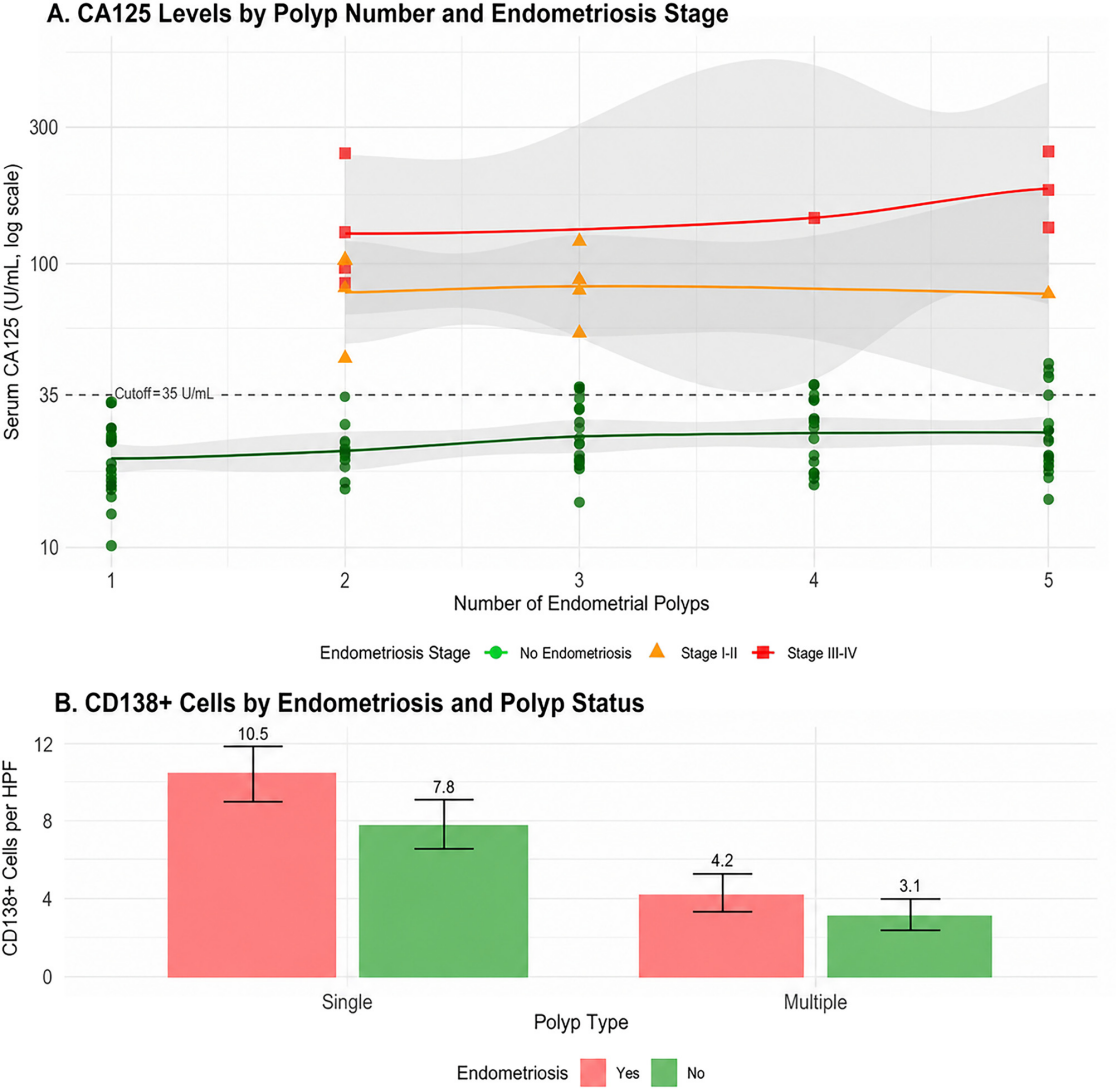
CA125 levels and chronic endometritis markers. (A) Serum CA125 levels by polyp number and endometriosis stage. Horizontal line indicates 35 U/mL cutoff. (B) CD138+ cell counts per HPF in patients with single versus multiple polyps, stratified by endometriosis status. Error bars show SEM.

Chronic endometritis, defined by CD138 ≥ 5 cells/HPF, was present in 63.6 % of women with EM compared to 25.0 % without EM (P = 0.015). The combination of CE and multiple polyps was found in 46.2 % of the EM group versus 9.2 % of the non-EM group (P = 0.002). Sensitivity analyses using different CD138 thresholds confirmed the robustness of this association (Figure 2).

### 3.5 Multivariate analysis and predictive model

Given the small number of EM events (*n* = 13), we employed Firth’s penalised logistic regression to obtain stable estimates and avoid overfitting (Table 5). The final model was limited to three predictor variables to maintain adequate events per variable.

**Table 5:** Univariate and multivariate logistic regression analysis for predicting endometriosis.

Variable	Univariate analysis		Multivariate analysis (Firth’s penalized)	
	OR (95 % CI)	*p*-Value	Adjusted OR (95 % CI)	*p*-Value
**Clinical factors**				
Age <35 years	4.82 (1.43–16.21)	0.011	–	–
BMI <25 kg/m^2^	3.15 (0.84–11.85)	0.089	–	–
Nulliparity	4.33 (1.13–16.56)	0.032	–	–
Primary infertility	4.22 (1.26–14.13)	0.019	–	–
**Symptoms**				
Dysmenorrhea (any)	15.48 (3.98–60.21)^a^	<0.001	8.45 (2.34–30.52)	0.001
Severe dysmenorrhea	28.58 (5.82–140.35)^a^	<0.001	–	–
Chronic pelvic pain	12.75 (3.55–45.78)	<0.001	–	–
Deep dyspareunia	7.44 (1.99–27.86)	0.003	–	–
**Polyp characteristics**				
Multiple polyps	3.05 (1.02–9.12)	0.046	3.12 (1.08–9.01)	0.036
Cornual location	3.89 (1.14–13.28)	0.030	–	–
Multiple locations	3.38 (1.08–10.56)	0.036	–	–
**Laboratory markers**				
CA125 > 35 U/mL	29.63 (7.31–120.12)^a^	<0.001	12.67 (3.82–42.01)	<0.001
CA125 (continuous, per 10 U/mL)	1.84 (1.42–2.38)	<0.001	–	–
**Pathological findings**				
CD138 positive (≥5 cells/HPF)	5.16 (1.38–19.31)	0.015	–	–

^a^Net Reclassification Improvement compared to CA125 alone.

Standard logistic regression produced wide confidence intervals due to quasi-separation

The final multivariate model identified 3 independent predictors of EM: dysmenorrhoea (adjusted OR 8.45, 95 % CI: 2.34–30.52, P = 0.001), CA125 > 35 U/mL (adjusted OR 12.67, 95 % CI: 3.82–42.01, P < 0.001), and multiple polyps (adjusted OR 3.12, 95 % CI: 1.08–9.01, P = 0.036). Bootstrap validation (1,000 resamples) yielded similar estimates with slightly wider confidence intervals, confirming model stability.

**Figure 3:**
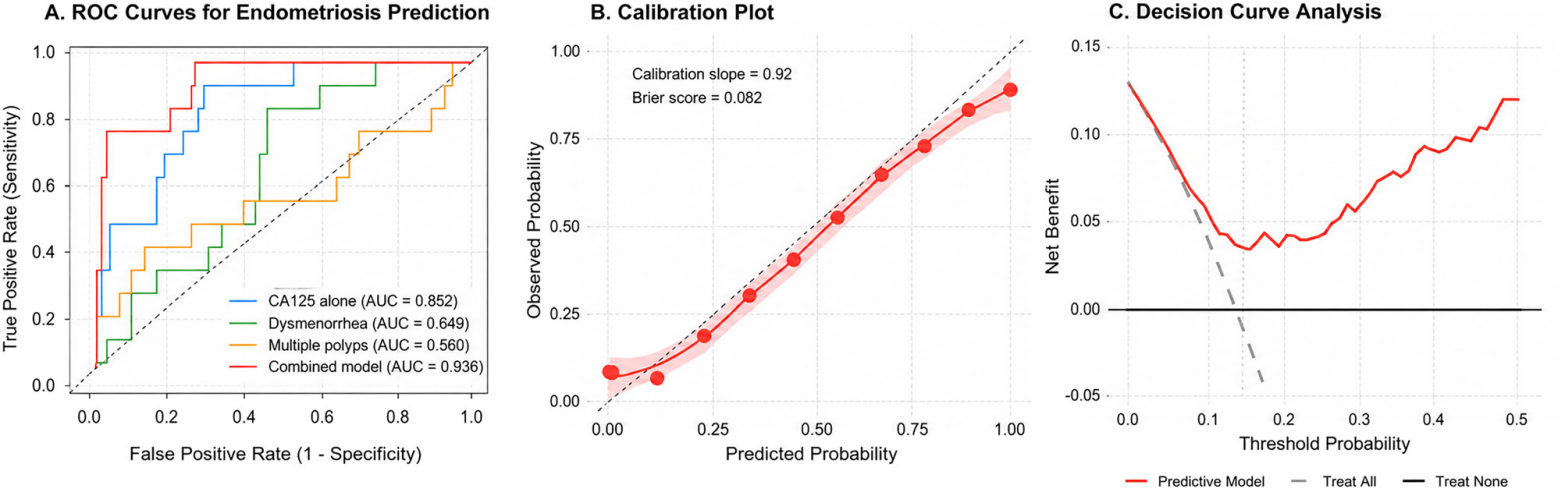
Diagnostic performance of predictive models. (A) ROC curves comparing CA125 alone (AUC = 0.852), dysmenorrhea (AUC = 0.649), multiple polyps (AUC = 0.560), and combined model (AUC = 0.938) for predicting endometriosis. (B) Calibration plot showing predicted versus observed probabilities. (C) Decision curve analysis demonstrating net benefit across threshold probabilities.

The combination of CA125 > 35 U/mL and dysmenorrhoea achieved the best diagnostic performance with 93 % accuracy (Table 6). The final predictive model showed excellent discrimination with an apparent AUC of 0.938 and a bootstrap-corrected AUC of 0.925, indicating minimal optimism (Figure 3).

**Table 6:** Diagnostic performance of combined markers for endometriosis detection.

Marker/combination	Sensitivity (%)	Specificity (%)	PPV (%)	NPV (%)	Accuracy (%)	NRI^a^
CA125 > 35 U/mL	76.9	89.7	52.6	96.3	88.0	Reference
Dysmenorrhea	100.0	64.4	29.5	100.0	69.0	−0.19
Multiple polyps	69.2	57.5	19.6	92.6	59.0	−0.29
CA125 + dysmenorrhea	76.9	95.4	71.4	96.5	93.0	+0.05
CA125 + multiple polyps	53.8	96.6	70.0	93.3	91.0	+0.03
All three markers	38.5	98.9	83.3	90.5	90.0	+0.02
Predictive model (all three markers)	84.6	89.7	55.0	97.5	89.0	+0.01

Abbreviations: NPV, negative predictive value; NRI, net reclassification improvement; PPV, positive predictive value. ᵃNRI was calculated using CA125 > 35 U/mL as the reference marker. Positive values indicate improved reclassification compared with CA125 alone, whereas negative values indicate poorer reclassification.

### 3.6 Fertility outcomes and follow-up

Among the 63 women who attempted to conceive during follow-up (out of the total 100 patients), complete fertility outcome data were available for 11 with EM and 52 without EM (Table 7). The median follow-up for fertility outcomes was 24 months (IQR 18–36 months).

**Table 7:** Fertility outcomes and pregnancy rates.

Outcome	Endometriosis (*n* = 11)	No endometriosis (*n* = 52)	*p*-Value	RR (95 % CI)
**Natural conception**				
Attempting pregnancy	11 (100)	52 (100)	–	–
Clinical pregnancy, n (%)	3 (27.3)	28 (53.8)	0.112	0.51 (0.18–1.40)
Time to pregnancy (months), median	14.5 (8–22)	8.2 (4–15)	0.038	–
Biochemical pregnancy, n (%)	5 (45.5)	32 (61.5)	0.327	0.74 (0.36–1.51)
Miscarriage, n (%)	1/3 (33.3)	4/28 (14.3)	0.412	2.33 (0.33–16.51)
Live birth, n (%)	2 (18.2)	24 (46.2)	0.103	0.39 (0.11–1.44)
**ART outcomes**				
Underwent ART, n (%)	8 (72.7)	31 (59.6)	0.511	–
**Intrauterine insemination (IUI) outcomes**	*n* = 5	*n* = 18		
Total IUI cycles	12	48	–	–
Cycles per patient, mean	2.4	2.7	0.642	–
Clinical pregnancy per patient	1/5 (20.0)	7/18 (38.9)	0.633	0.51 (0.08–3.41)
Clinical pregnancy per cycle	1/12 (8.3)	7/48 (14.6)	0.681	0.57 (0.08–4.25)
***In vitro* fertilization (IVF)/intracytoplasmic sperm injection (ICSI) outcomes**	*n* = 8	*n* = 31		
Total cycles performed	15	42	–	–
Cycles per patient, mean	1.9	1.4	0.182	–
Oocytes retrieved, mean	8.2 ± 4.1	10.5 ± 5.3	0.156	–
Fertilization rate (%)	68.3	72.1	0.423	–
Clinical pregnancy per transfer	4/15 (26.7)	18/42 (42.9)	0.271	0.62 (0.24–1.61)
Implantation rate	6/28 (21.4)	24/86 (27.9)	0.494	0.77 (0.35–1.69)
Live birth per patient	3/8 (37.5)	15/31 (48.4)	0.703	0.78 (0.29–2.08)

Kaplan–Meier analysis of time to pregnancy showed that women with EM had significantly longer time to conception (log-rank P = 0.041), with a 12-month cumulative pregnancy rate of 27.3 % versus 53.8 % in the non-EM group.

### 3.7 Polyp recurrence during follow-up

Polyp recurrence data were available for 79 patients who underwent complete polyp removal and had at least one follow-up evaluation (Table 8). The median follow-up duration was 17.5 months (range 6–42 months).

**Table 8:** Polyp recurrence after hysteroscopic polypectomy.

Parameter	Endometriosis (*n* = 11)^a^	No endometriosis (*n* = 68)^a^	*p*-Value	HR (95 % CI)^b^
**Follow-up characteristics**				
Follow-up duration (months), median (IQR)	18.2 (12–24)	16.8 (12–24)	0.543	–
Lost to follow-up before 12 months	2/13 (15.4)	19/87 (21.8)	0.732	–
**Recurrence outcomes**				
Any recurrence, n (%)	5 (45.5)	12 (17.6)	0.047	3.12 (1.09–8.91)
Time to recurrence (months), median	8.5 (6–14)	11.2 (8–18)	0.236	–
**Cumulative recurrence rates**				
6 months, % (95 % CI)	18.2 (2.3–44.2)	4.4 (1.1–11.2)	0.082	–
12 months, % (95 % CI)	36.4 (10.9–62.7)	10.3 (4.2–19.2)	0.024	–
18 months, % (95 % CI)	45.5 (16.7–70.7)	16.2 (8.2–26.4)	0.019	–
24 months, % (95 % CI)	45.5 (16.7–70.7)	17.6 (9.2–28.1)	0.031	–
**Recurrence by initial presentation**				
Single polyp → recurrence	1/3 (33.3)	4/38 (10.5)	0.319	–
Multiple polyps → recurrence	4/8 (50.0)	8/30 (26.7)	0.225	–
**Recurrence by CE status**				
CE positive → recurrence	4/6 (66.7)	7/15 (46.7)	0.633	–
CE negative → recurrence	1/5 (20.0)	5/53 (9.4)	0.436	–
**Management of recurrence**				
Repeat hysteroscopy	4 (80.0)	8 (66.7)	1.000	–
Medical management only	1 (20.0)	3 (25.0)	1.000	–
Expectant management	0 (0)	1 (8.3)	1.000	–

^a^Patients with complete follow-up data after polypectomy; ^b^Hazard ratio from Cox regression.

**Figure 4:**
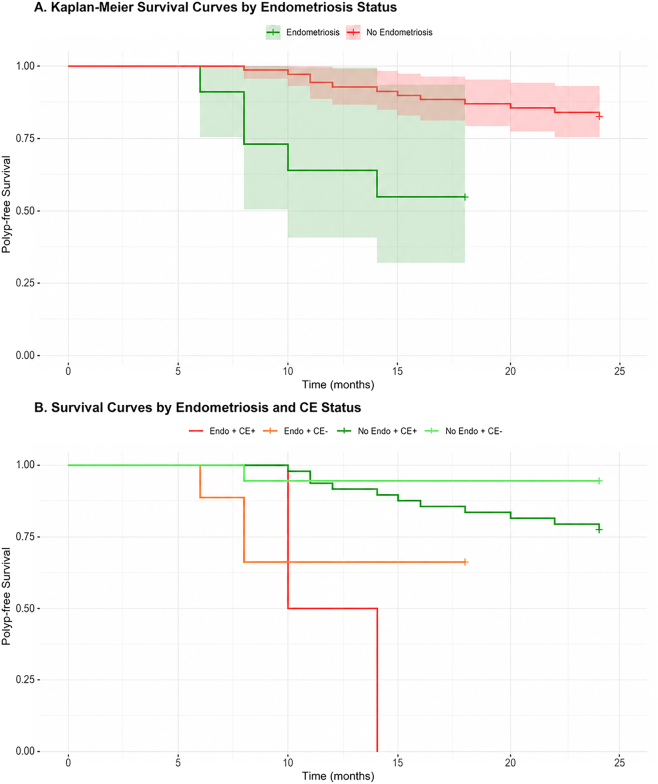
Polyp recurrence-free survival analysis. (A) Kaplan-Meier curves comparing polyp-free survival between patients with (*n* = 11) and without (*n* = 68) endometriosis (log-rank p = 0.022). (B) Survival curves stratified by both endometriosis and chronic endometritis (CE) status. Shaded areas represent 95 % confidence intervals.

The overall recurrence rate was significantly higher in women with EM (45.5 % vs. 17.6 %, P = 0.047). Kaplan–Meier survival analysis showed significantly shorter recurrence-free survival in women with EM (log-rank P = 0.022), with a hazard ratio of 3.12 (95 % CI: 1.09–8.91) after adjustment for age and initial polyp number (Figure 4).

### 3.8 Sensitivity analyses

Several sensitivity analyses were performed to assess the robustness of our findings. Because adenomyosis shares clinical features with EM (including dysmenorrhoea and elevated CA125) and could potentially confound the analysis, we performed a sensitivity analysis excluding the 4 women with adenomyosis to assess whether the observed associations were driven solely by those with pure EM. When these 4 patients were excluded, leaving 9 with confirmed EM only, the associations remained significant for dysmenorrhoea (OR 7.82, P = 0.003), CA125 (OR 10.54, P < 0.001), and multiple polyps (OR 2.89, P = 0.048), indicating that the presence of adenomyosis did not drive the main findings. Using CD138 ≥ 1 cell/HPF as the threshold for CE increased the prevalence to 81.8 % in EM versus 44.4 % in non-EM groups (P = 0.027). Using ≥10 cells/HPF decreased prevalence, but maintained the significant association. In women <40 years (*n* = 42), the association between EM and multiple polyps was stronger (OR 4.12, P = 0.021), while in women ≥40 years (*n* = 58), the association was not significant (OR 2.14, P = 0.234). After imputing missing laboratory values using multiple imputation (5 datasets), the results remained consistent with complete case analysis.

## 4 Discussion

This comprehensive retrospective cohort study provides robust evidence for a significant association between EPs and EM, with important implications for clinical management and our understanding of endometrial pathophysiology. Our primary finding that 13 % of women with EPs have concurrent EM aligns with recent meta-analyses reporting prevalence rates of 10 %–20 % in similar populations [1], 10]. This consistency across studies suggests that the coexistence of EPs and EM is unlikely to be incidental. Instead, it may reflect shared pathogenic mechanisms involving oestrogen-dependent proliferation and chronic inflammatory activation within the endometrium [11]. More importantly, we identified 3 independent predictors of EM among women with polyps: dysmenorrhoea (adjusted OR 8.45), elevated CA125 > 35 U/mL (adjusted OR 12.67), and multiple polyps (adjusted OR 3.12). These findings provide clinically actionable markers for risk stratification that could guide surgical planning and patient counselling.

While the 13 % prevalence of EM in this cohort may appear modest, this rate is notable given that all patients were initially diagnosed with EPs, and reflects the enriched prevalence in a surgical population compared to the estimated 2 %–10 % prevalence in the general female population [12]. More importantly, the strength of the association lies not in this crude prevalence, but in the identification of independent predictors (dysmenorrhoea OR 8.45, CA125 OR 12.67, multiple polyps OR 3.12) that were highly significant despite the small number of events, underscoring the robustness of these clinical markers.

The universal presence of dysmenorrhoea in our EM cohort (100 % vs. 35.6 % in non-EM) represents one of the strongest clinical associations identified to date. While this may partly reflect selection bias in a surgical population, the severity gradient observed (53.8 % with severe dysmenorrhoea in EM vs. 3.4 % without) suggests a genuine biological relationship. This finding has immediate clinical utility: women presenting with EPs and significant dysmenorrhoea should be counselled about the possibility of concurrent EM. Recent clinical studies have similarly highlighted dysmenorrhoea as one of the most consistent clinical indicators of underlying EM in women undergoing gynaecologic surgery [11]. This approach could reduce diagnostic delay, which currently averages 7–10 years for EM [13], and enable comprehensive treatment in a single surgical session.

The markedly elevated CA125 levels observed in our EM cohort (mean 89.3 U/mL) extend previous findings by demonstrating utility specifically in the context of EPs. Although CA125 alone has limited sensitivity for screening in the general population, recent studies suggest it remains a useful adjunct biomarker reflecting peritoneal inflammation and disease burden in EM [11]. The diagnostic accuracy achieved exceeds that reported in general populations, likely due to the enriched prevalence of EM in women with polyps [5], 14]. Importantly, the combination of CA125 with clinical symptoms improved diagnostic performance, achieving 93 % accuracy when combined with dysmenorrhoea. This multimodal approach addresses the limitations of using CA125 alone and could form the basis of a clinical decision tool for surgical planning.

Our findings both confirm and extend previous observations regarding the EP–EMs association. A systematic review by Vitagliano et al. [7]. found that women with EM had a 2.81-fold higher risk of EPs (95 % CI: 2.48–3.18), while our study provides a complementary perspective: among women with confirmed polyps, those with EM had distinct clinical and pathological features. The higher prevalence of multiple polyps in our EM cohort (69.2 % vs. 42.5 %) supports the hypothesis that systemic inflammatory and oestrogen-dependent signalling associated with EM may promote multifocal endometrial proliferation [1].

Our novel finding of increased polyp prevalence in cornual locations (38.5 % vs. 13.8 %, P = 0.041) and multiple sites (61.5 % vs. 32.2 %, P = 0.036) in women with EM has not been previously reported and may reflect altered endometrial receptivity or local inflammatory changes near the tubal ostia.

This observation has potential implications for fertility, given that cornual polyps can significantly impede sperm transport or embryo implantation [15]. The clinical relevance is underscored by our fertility outcomes showing longer time to pregnancy and lower clinical pregnancy rates in the EM group, though larger studies are needed to confirm these associations.

The high prevalence of CE in our EM cohort (63.6 % vs. 25.0 %, P = 0.015) represents an important finding linking two previously independent areas of research. A recent prospective study further demonstrated that CE significantly increased the recurrence risk of EPs following hysteroscopic resection [16], but our study is among the first to examine this triad comprehensively. The synergistic effect observed (women with EM, multiple polyps, and CE having the highest recurrence rates) suggests that these conditions may share common pathophysiological mechanisms involving chronic inflammation, altered immunity, and microbial dysbiosis.

The strong association between EPs and EM likely reflects multiple interconnected pathophysiological mechanisms. Both conditions are oestrogen-dependent, and the local hyperoestrogenic environment created by endometriotic lesions through aromatase expression may promote polyp formation [17]. Our finding that women with EM were younger (32.5 vs. 43.2 years) yet had a similar or higher polyp burden suggests accelerated endometrial proliferation in the setting of EM, possibly due to prolonged oestrogen exposure or increased endometrial sensitivity to hormonal stimulation.

The inflammatory hypothesis is supported by multiple lines of evidence from our study. The high prevalence of CE, elevated CA125 levels, and the universal presence of dysmenorrhoea in the EM group all point to a chronic inflammatory state. Recent molecular studies have identified elevated levels of inflammatory cytokines, including interleukin-6, tumour necrosis factor-alpha, and vascular endothelial growth factor in both endometriotic lesions and EPs [2], 18]. These inflammatory mediators can promote angiogenesis, cell proliferation, and resistance to apoptosis – processes that are central to both polyp formation and EM progression. Our observation of increased vascularity in polyps from women with EM (though not statistically significant) aligns with this enhanced angiogenic activity.

The emerging role of the endometrial microbiome may provide another potential mechanistic link. Recent microbiome studies have reported that vaginal and endometrial dysbiosis may contribute to abnormal endometrial proliferation and increase the recurrence risk of EPs after hysteroscopic removal [13]. Recent 16S ribosomal ribonucleic acid sequencing studies have demonstrated dysbiosis in women with both conditions, characterised by reduced Lactobacillus dominance and increased bacterial diversity [16], 19]. This altered microbiome may trigger chronic inflammation through the activation of toll-like receptors, leading to plasma cell infiltration (CE) and creating a permissive environment for both polyp formation and endometriotic implant survival. The observation that women with CE had higher polyp recurrence rates supports this microbial–inflammatory hypothesis and suggests that treating the underlying dysbiosis could improve outcomes. This observation is consistent with recent clinical evidence showing that CE can significantly increase the recurrence risk of EPs [16].

Genetic and epigenetic factors likely contribute to the EP–EMs association. Recent genomic studies have identified somatic mutations in cancer driver genes, including KRAS, PIK3CA, and ARID1A in both EPs and endometriotic lesions [20], 21]. These mutations, while not conferring malignancy, may promote clonal expansion and abnormal proliferation. The presence of similar mutations in both conditions suggests they may represent different manifestations of a field effect affecting the entire reproductive tract, possibly initiated by common environmental exposures or hormonal influences during critical developmental windows.

Our findings have several clinical implications for improving the diagnosis and management of women with EPs and EM. Measuring CA125 in women with polyps, especially those with dysmenorrhoea or who are infertile, can aid diagnostic workup, with CA125 > 35 U/mL showing high specificity; adjunct imaging with transvaginal ultrasound or magnetic resonance imaging may further enhance detection. Fertility counselling is crucial, as these women face a longer time to pregnancy and lower success rates, warranting comprehensive evaluation and early assisted reproduction, particularly for those >35 years. Surveillance should also be intensified, given the higher polyp recurrence in the EM group (45.5 % vs. 17.6 %), with a 6-monthly ultrasound in the first 2 years and consideration of hormonal suppression for those not pursuing pregnancy [22], 23]. Finally, given the frequent coexistence of CE, CD138 immunohistochemistry, and antibiotic therapy (e.g. doxycycline 100 mg bid for 14 days) may improve outcomes, though dedicated trials in this subgroup are still needed [24], 25].

This study has notable strengths, including comprehensive surgical evaluation with hysteroscopy and laparoscopy plus histopathology for definitive diagnosis, the use of Firth’s penalised logistic regression to address small event numbers, incorporation of diverse clinical and laboratory parameters, prospective follow-up assessing fertility and recurrence, and sensitivity analyses confirming robustness.

However, limitations include its retrospective single-centre design with potential selection bias, a small number of EM cases (*n* = 13) limiting subgroup analyses and the precision of estimates, an inability to establish temporal relationships, incomplete evaluation for CE, variable laboratory data, the absence of molecular or microbiome profiling, and a relatively short follow-up that may underestimate long-term outcomes. Future research should focus on large prospective cohorts to clarify causality, molecular and microbiome studies to explore mechanisms, randomised trials to optimise surgical strategies, adjuvant treatments and CE management, the development of predictive biomarkers, and the long-term surveillance of fertility and cancer risk. Clinically, current guidelines may require revisions to reflect the frequent co-occurrence of polyps and EM, incorporating CA125 and symptom assessment into diagnostic algorithms, considering concurrent laparoscopy in high-risk women, adopting risk-stratified follow-ups, and providing fertility management recommendations tailored to this population. The retrospective study design limited our ability to establish causal relationships, and the relatively small number of EM cases (*n* = 13) may have reduced the statistical power for some subgroup analyses. Additionally, while our sensitivity analysis suggested that the exclusion of adenomyosis cases did not alter the main findings, the small number of patients with concurrent adenomyosis (*n* = 4) precluded definitive conclusions about its independent effect.

## 5 Conclusions

This retrospective cohort study of 100 women with EPs undergoing concurrent hysteroscopy and laparoscopy found that dysmenorrhoea, CA125 > 35 U/mL, and multiple polyps emerged as strong independent predictors of concurrent EM. Women with both conditions were younger, had a greater symptom burden, higher rates of CE, poorer fertility outcomes, and increased polyp recurrence, suggesting a more aggressive phenotype requiring comprehensive evaluation and individualised management.
